# The Evolution of Senescence and Post-Reproductive Lifespan in Guppies *(Poecilia reticulata)*

**DOI:** 10.1371/journal.pbio.0040007

**Published:** 2005-12-27

**Authors:** David Reznick, Michael Bryant, Donna Holmes

**Affiliations:** **1**Department of Biology and Center for Conservation Biology, University of California Riverside, Riverside, California, United States of America; **2**School of Critical Studies, California Institute of the Arts, Valencia, California, United States of America; **3**Department of Biological Sciences, University of Idaho, Moscow, Idaho, United States of America; University of Newcastle upon TyneUnited Kingdom

## Abstract

The study of post-reproductive lifespan has been of interest primarily with regard to the extended post-menopausal lifespan seen in humans. This unusual feature of human demography has been hypothesized to have evolved because of the “grandmother” effect, or the contributions that post-reproductive females make to the fitness of their children and grandchildren. While some correlative analyses of human populations support this hypothesis, few formal, experimental studies have addressed the evolution of post-reproductive lifespan. As part of an ongoing study of life history evolution in guppies, we compared lifespans of individual guppies derived from populations that differ in their extrinsic mortality rates. Some of these populations co-occur with predators that increase mortality rate, whereas other nearby populations above barrier waterfalls are relatively free from predation. Theory predicts that such differences in extrinsic mortality will select for differences in the age at maturity, allocation of resources to reproduction, and patterns of senescence, including reproductive declines. As part of our evaluation of these predictions, we quantified differences among populations in post-reproductive lifespan. We present here the first formal, comparative study of the evolution of post-reproductive lifespan as a component of the evolution of the entire life history.

Guppies that evolved with predators and that experienced high extrinsic mortality mature at an earlier age but also have longer lifespans. We divided the lifespan into three non-overlapping components: birth to age at first reproduction, age at first reproduction to age at last reproduction (reproductive lifespan), and age at last reproduction to age at death (post-reproductive lifespan). Guppies from high-predation environments live longer because they have a longer reproductive lifespan, which is the component of the life history that can make a direct contribution to individual fitness. We found no differences among populations in post-reproductive lifespan, which is as predicted since there can be no contribution of this segment of the life history to an individual's fitness.

Prior work on the evolution of post-reproductive lifespan has been dominated by speculation and correlative analyses. We show here that this component of the life history is accessible to formal study as part of experiments that quantify the different segments of an individual's life history. Populations of guppies subject to different mortality pressures from predation evolved differences in total lifespan, but not in post-reproductive lifespan. Rather than showing the direct effects of selection characterizing other life-history traits, post-reproductive lifespan in these fish appears to be a random add-on at the end of the life history. These findings support the hypothesis that differences in lifespan evolving in response to selection are confined to the reproductive lifespan, or those segments of the life history that make a direct contribution to fitness. We also show, for the first time, that fish can have reproductive senescence and extended post-reproductive lifespans despite the general observation that they are capable of producing new primary oocytes throughout their lives.

## Abstract 2

An analysis of the causes of variation in post-reproductive lifespan reveals that fish senesce and that the evolution of lifespan in guppies is due to selection during their reproductive stage.

## Introduction

The post-reproductive segment of the human lifespan is often considered to be an adaptive feature of aging, since post-reproductive women can make significant contributions to the fitness of their children and grandchildren [1,2]. Although there is published evidence for the adaptive significance of the post-reproductive life stage in humans [3], few formal empirical analyses have been conducted to conclusively demonstrate that this segment of the life history has been shaped by natural selection. Evolutionary theory predicts that an extended post-reproductive lifespan should evolve only when post-reproductive females can contribute significantly to the fitness of their offspring or relatives. Such fitness contributions can occur only when post-reproductive maternal care is required for the last young that mothers produce [4], or when animals have extended social networks allowing post-reproductive females to contribute significantly to the fitness of close relatives, as in humans [5,6]. Where such kin networks exist, any advantage of post-reproductive lifespan must also be sufficient to offset the waning force of natural selection associated with the low probability of females surviving to advanced ages [5,6]. If no such post-reproductive contribution to fitness exists, then any post-reproductive lifespan that we see should represent a variable and random segment of the life history incidental to differences in aging rates of reproductive and somatic tissues.

The phenomenon of post-reproductive lifespan is by no means limited to human females, or even to female primates. Midlife cessation of ovulation, followed by a post-reproductive lifespan of up to a third of the reported species maximum lifespan, has been reliably documented in a wide range of female vertebrates in captivity or under other favorable conditions. These species include guppies *(Poecilia reticulata)* [7], platyfish [8], Japanese quail [9,10,11], budgerigar “parakeets” [12], laboratory rats and mice [13,14,15], opossums[16], and primates. [17,18,19]. Most of these animals lack well-developed kin networks and engage in very limited, if any, maternal care. In some (e.g., platyfish and Japanese quail), males also exhibit midlife loss of reproductive capacity [8,20,11]. These observations suggest that, rather than being a result of kin selection, midlife fertility loss and post-reproductive lifespan may be correlates of extended lifespan under particularly favorable conditions characterized by good nutrition and unusually low rates of mortality from parasites, predators, disease, and accident.

The strongest evidence for the adaptive significance of post-reproductive lifespan in humans comes from historical, multigenerational demographic records for 18th and 19th century populations in Canada and Finland [3]. In analyses of data of this type, survival of grandmothers has been associated with improved reproductive success in their offspring. These analyses also suggest that this “grandmother effect” requires active behavioral interactions between older females and kin. The effect was detected only when grandmothers lived in the same town as kin receiving the benefit and accrued only by grandchildren that survived past weaning. The positive effect of grandmothering was present even after controlling statistically for potentially confounding factors, such as socioeconomic status, temporal trends in survival, or geography.

Comparable analyses have been performed for other species. Packer et al. [4] report on baboons and lions, both of which live in extended family groups in which grandmothers have extended interactions with kin. In both species, however, females generally live only long enough to care for their last-born offspring, and no positive effect of grandmothers' post-reproductive survival was detected. Female salmon, like lions and baboons, live long enough after spawning to enhance the fitness of their offspring. They prevent the nest site from being reused by late-arriving spawners; such reuse causes a substantial reduction in the viability of the eggs [21].

We have evaluated the pre-reproductive, reproductive, and post-reproductive lifespan in guppies in relation to selective regimes shaping the evolution of their early life histories and patterns of senescence. We compared life-history traits in guppies from high- versus low-predation environments in Trinidad. Guppies from high-predation environments co-occur with predators that frequently prey on guppies, and particularly on large, adult-size classes. Guppies from low-predation environments co-occur with just a single species of fish, *Rivulus hartii,* that feeds on guppies only occasionally, and tends to feed on small, immature-size classes. These two types of localities are often found in the same drainage in close proximity of one another, separated by barrier waterfalls that exclude larger predators but not guppies or *Rivulus*.

In nature, guppies from high-predation localities sustain higher mortality rates than their counterparts from low-predation localities [22]. Furthermore, guppies from high-predation localities attain maturity at an earlier age, produce more offspring per litter, and reproduce more often than guppies from low predation localities [23,24], which is the predicted evolutionary response to higher adult mortality rates [6]. In addition, we have manipulated the mortality rates that guppies experience in nature with guppy or predator introduction experiments and have shown that these life history patterns can evolve in relatively brief intervals of time [25,26,27]. We have also documented senescence in natural populations in the form of an acceleration of mortality rate with age [28].

We compared the patterns of aging and senescence in the second laboratory-born generation of guppies from high- versus low-predation environments. We evaluated guppies from the Yarra and Oropuche drainages and included a high- and low-predation environment from each drainage, for a total of four populations. Since genetic data show that guppies adapted to these regimes independently in each locality [29,30], this approach provides duplicate studies of adaptation of guppy populations to the presence or absence of predators. Classical evolutionary senescence theory predicts that animals from high-predation environments will experience earlier or more rapid senescence than those from low-predation environments, either as a byproduct of intense selection for increased investment in reproduction early in life or because of the accumulation of deleterious mutations affecting older individuals [31,32]. We reported previously that the early life histories of high-predation guppies in this experiment were different from those from low-predation localities in the same drainage in a fashion that is consistent with our earlier comparisons [33]: They matured earlier, produced more young per litter, and reproduced more frequently. Contrary to expectation, however, we found that when guppies were reared in the lab free from predation, those from high-predation environments have lower initial mortality rates, lower mortality rates throughout their lives, and longer median lifespans [34]. Furthermore, guppies from high-predation localities have higher fecundity throughout their lives.

Here we consider in more detail why guppies from high-predation environments have longer median lifespans and, specifically, whether the length of post-reproductive lifespan varies in guppies that have evolved under different mortality regimes. Life-history theory predicts that selection will shape only segments of the life history that contribute directly to fitness or are correlated with other life-history variables. Specifically, selection should favor the evolution of an extended post-reproductive lifespan only if post-reproductive individuals can contribute to their inclusive fitness in some way, either by caring for their own young, other kin, or grand-offspring. Since guppies are livebearers and provide no parental care after birth, we predicted that there should be no direct selection for an extended post-reproductive lifespan in this species. Any post-reproductive lifespan we observe, therefore, should represent a by-product of different aging rates of different parts of the soma or, alternatively, a correlate of selection for traits—including reproductive characteristics—that are adaptive earlier in the life history.

Unlike birds and mammals, which produce the vast majority of oocytes before birth, fish are generally believed to produce new oocytes throughout their lives. For this reason, fish have been suggested to show little or no reproductive senescence [35]. The empirical evidence for such unlimited reproductive capacity, however, is based on isolated observations of very old, fertile individuals and a few small-scale laboratory studies of senescence in fish; few comparative studies have directly addressed reproductive aging in fish. Here we formally evaluate, for the first time, whether or not there is reproductive senescence with an extended post-reproductive lifespan in a species of fish.

## Results

### Life-History Segments

We have previously shown that guppies from high-predation environments in this study are younger at maturity than those from low-predation environments [33], and that these high-predation guppies have lower rates of loss of reproductive function, lower mortality rates throughout their lives, and longer median lifespans [34].

We first present analyses of variance (ANOVA) of the three, non-overlapping segments of the lifespan: age at first reproduction, reproductive lifespan (age at last reproduction minus age at first reproduction), and post-reproductive lifespan (age at death minus age at last reproduction). As we found earlier, guppies from high-predation localities are younger at first reproduction. Their reproductive lifespan is also significantly longer, enough for them to be far older, on average, when they cease reproduction. Furthermore, reproductive lifespan is longer in guppies from the Oropuche drainage than in the Yarra drainage and in guppies reared on low as opposed to high levels of food availability (Tables 1 and 2). Low food caused a much larger increase in the reproductive lifespan of guppies from low- as opposed to high-predation localities, which in turn resulted in a marginally significant interaction between food availability and predation. There were no differences among any treatment groups for the duration of post-reproductive lifespan.

**Table 1:**
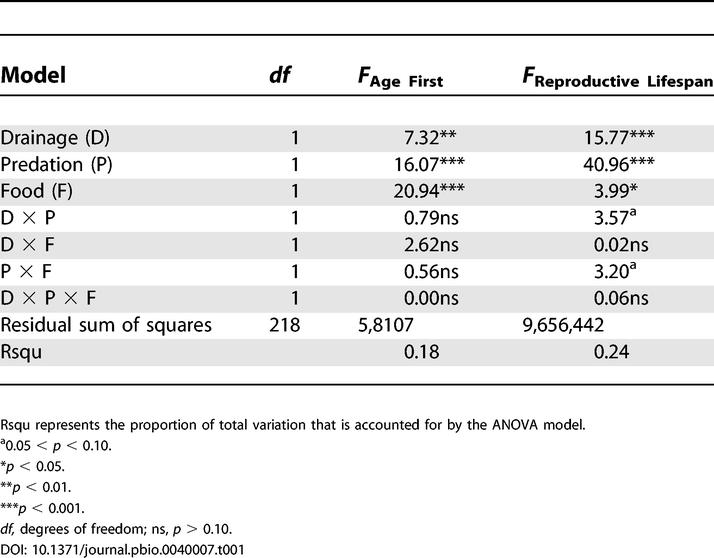
Summary of ANOVAs for the Age at First Reproduction and the Duration of the Reproductive Lifespan

**Table 2:**
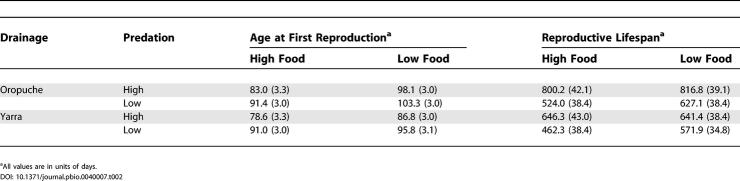
Least-Square Means (Standard Error) from the ANOVAs in Table 1

### Interbrood Interval

The inference that an individual has a significant post-reproductive lifespan is based on an analysis of each individual's frequency of reproduction. For each individual, we asked whether or not it had lived beyond the time interval in which it could be expected to produce another litter of young. Fish usually reproduced at regular intervals of 22–35 days and produced an average of 15 to 28 litters of young during their lives (Figure 1, Table 3). As they aged, some individuals appeared to skip litters or even cease reproducing for an interval of time, then resume reproduction. This irregular cessation of reproduction accounts for the unusually long interbrood intervals, sometimes in excess of 100 d, seen in the figure. Sample lifetime reproductive schedules for three individuals from one treatment group (Figure 1, bottom) illustrate some of the individual variation that we saw in this study. One individual reproduced at regular intervals throughout its life, then died before the age when it could be expected to produce a new litter of young. A second individual reproduced less regularly and appears to have skipped what would have been its fifth and eighth litters, then died a few days after its last litter was born. Neither of these fish showed evidence of having lived past the time when they might have produced an additional litter of young, so neither was scored as having a significant post-reproductive lifespan. A third individual produced 22 broods of young at regular intervals, temporarily ceased reproduction, then produced five more litters at regular intervals. It then ceased reproduction and died long after the birth of its last brood. This individual was likely to have lived beyond its capacity to reproduce.

**Figure 1:**
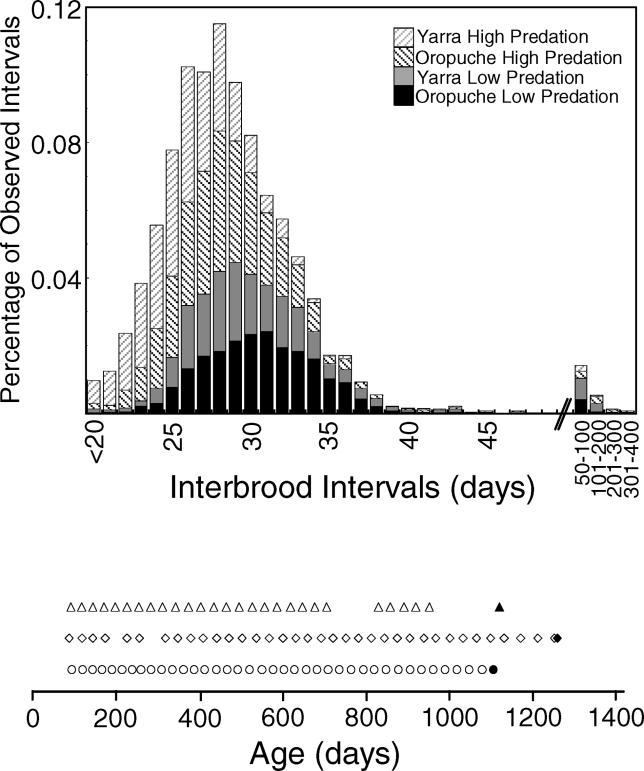
Interbrood Intervals Here we illustrate the frequency distribution of interbrood intervals for all individual intervals in the experiment. Vales are plotted for all litters less than 20 d, then per day for days 20–50. We then report the total number of litters for days 51–100, then in blocks per 100-d interval thereafter (e.g., values shown as “100” are 101–200). The bottom of the figure details the reproductive history of three individuals from the Oropuche high-predation treatment group to illustrate individual variation. Each individual's litter birth dates are represented by an open symbol on the *x*-axis, which represents the individuals age. The age at death is represented by a closed symbol. Details of these examples are described in the text.

**Table 3:**
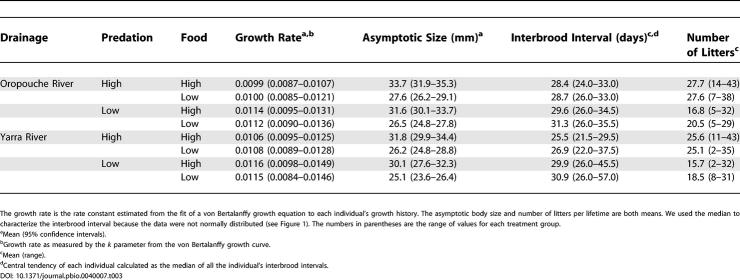
Values for Mean Growth Rate, Mean Asymptotic Body Size, Median Interbrood Interval, and Mean Number of Litters per Lifetime for Each Treatment Group

To accommodate this individual variation, we quantified the 90th percentile for the expected number of days between successive litters for each individual in the study, based on that individual's parturition records. We interpret living beyond that time interval as the equivalent of a one-tailed test of the probability that that individual lived beyond its capacity to produce an additional litter of young (see Materials and Methods). Individuals that died within that time interval might still have had the capacity to reproduce.

### Post-Reproductive Lifespan

Approximately 60% of the individuals in this study had post-reproductive lifespans exceeding their 90th percentile (Figure 2). We interpret this to mean that these individuals are likely to have lived beyond the time required for them to produce one more litter of young or that they had outlived their ability to reproduce.

**Figure 2:**
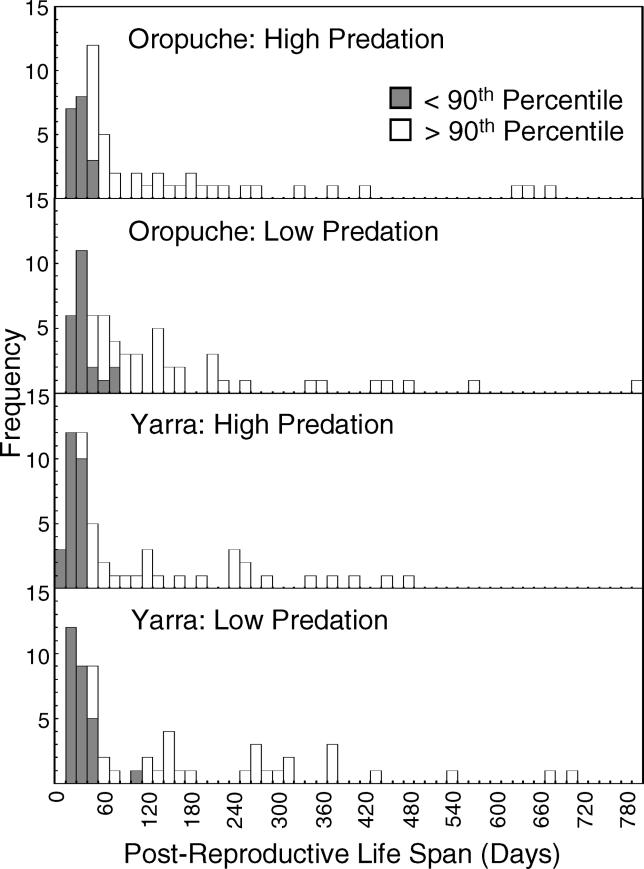
The Distribution of Post-Reproductive Lifespans Each drainage × predation combination is displayed separately. Filled bars represent females who died within the 90th percentile for their interbrood interval, or the interval during which they are expected to produce another brood of offspring. Open bars represent females that lived beyond their 90th percentile and hence are interpreted as having a significant post-reproductive lifespan.

We used logistic regression to ask whether or not there were differences among treatment groups in the probability of having a significant post-reproductive lifespan. The full model, which included drainage, food availability and predation, did not account for a significant proportion of the variability in the probability of living beyond an individual's 90th percentile (likelihood ratio Chi Square = 6.19, 3 df [degrees of freedom], *p* = 0.1027; data in Table 4). None of the three main effects were significant on their own (*p*_drainage_ = 0.0810, *p*_food_ = 0.0800, *p*_predation_ = 0.7058); the drainage effect was marginal because guppies from the Oropuche drainage were more likely to live beyond their 90th percentile than those from the Yarra drainage. We then evaluated the effects of food and predation within each drainage in four separate analyses. The effect of food was not significant within the Oropuche drainage (*p* = 0.61) and was marginally significant in the Yarra drainage (*p* = 0.0559); low-food fish were more likely to live beyond their 90th percentile. The effect of predation was not significant in either drainage (Yarra: *p* = 0.7097; Oropuche: *p* = 0.9784). Therefore, in spite of the substantial and significant differences among drainages and predation communities for other components of the life history, there were no significant differences among drainages or predation communities in either the duration of post-reproductive lifespan or the probability that an individual was likely to have ceased reproduction before dying.

**Table 4:**
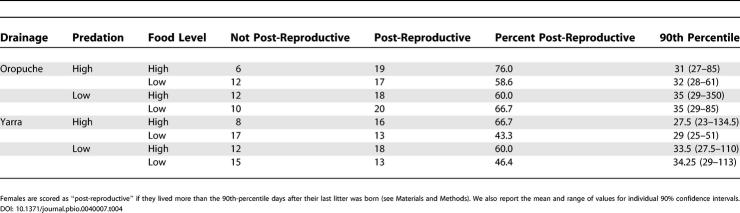
Number of Females with and without a Significant Post-Reproductive Lifespan

Comfort's [39] evaluation of reproduction in old, domestic guppies presents the only other data set for fish that we could find that includes information on post-reproductive lifespan, but it does not include any analyses. He only reports on the age at last reproduction and age at death, so we were only able to evaluate the distribution of the duration of post-reproductive lifespan for his fish. This distribution is similar in shape to our data and shows that 28 out of 36 fish lived beyond the age when they could be expected to produce another litter of young (Figure 3) if their variance in time intervals between successive litters is the same as in our fish.

**Figure 3:**
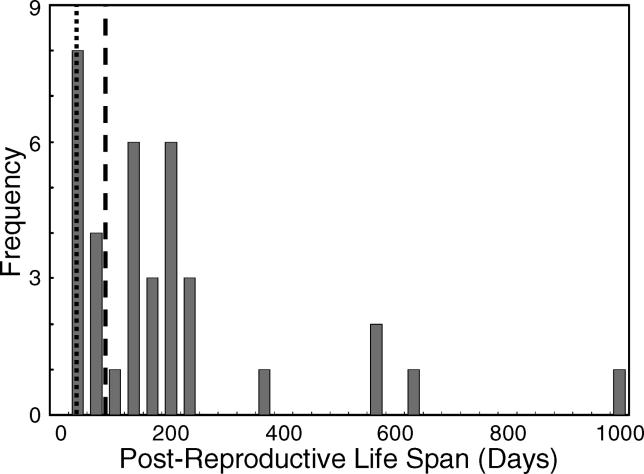
The Distribution of Values for Post-Reproductive Lifespan in Comfort (1961) Comfort [7] reported the age at last reproduction and age at death in one of his studies of senescence in domestic guppies. We report here a summary distribution of their post-reproductive lifespan. For purposes of comparison, the dotted vertical line represents the mean 90th percentile for the number of days between successive litters of young in our data set for Trinidadian guppies. The dashed line represents the 95th percentile for this mean value. This figure shows that the shape of the distribution of post-reproductive lifespans for Comfort's guppies was similar to that observed in our study. It also shows that the average duration of the post-reproductive lifespan was longer, which correlates positively with their longer total lifespans.

## Discussion

### Comparisons of Guppy Aging to Other Organisms

Our analyses show that guppies have a pattern of reproductive senescence, including significant post-reproductive lifespans, that is similar to that of many birds and mammals. We previously showed that there is an age-specific acceleration in mortality rate and decline in reproductive performance of guppies with age [34]. Here we have shown that most individuals also have an extended post-reproductive lifespan. The similarity between guppies and mammals in mortality was shown long-ago by Comfort [36], but comparable lifetime data for female reproduction are not available for any species of fish. Our findings refute the prediction that, because many fish appear to generate primary oocytes throughout adult life, fish will show negligible reproductive senescence [35]. Such predictions have been based on the observation of the production of oocytes in old, wild-caught fish [35,37] or fertility and presence of viable oocytes in some of the oldest representatives in laboratory studies of senescence on guppies [38,39] and platyfish and annual killifish [35].

From a quantitative perspective, we found that the median post-reproductive lifespan of guppies was 6.4% and the mean was 13.6% of the total lifespan, with a range of 0%–76% of the total lifespan. Investigators have reported post-reproductive lifespans of “up to” or “over” 30% of the total lifespan in birds and mammals [12,16], but these percentages tend to be reported as a single figure, rather than as being derived from a formal statistical analysis, as done here. Our data are thus not directly comparable to those of others, and we are not able to make direct comparisons between the duration of post-reproductive lifespans in guppies, birds, and mammals.

Mammals and birds produce the vast majority of primary oocytes during embryonic development [40,41]; this finite pool of eggs declines steadily during the lifespan. The usual explanation for midlife loss of ovulatory capacity in women and laboratory rodents is the depletion of this finite population of oocyte stores [42,43] however, recent work on mice [44] and humans [45] suggests that they retain primordial germ cells that continue to contribute to the pool of primary oocytes after birth. If guppies, like other fish, retain the capacity for producing new primary oocytes throughout adulthood, midlife cessation of ovulation in female fish, birds, or mammals may not always be dictated by the extinction of a finite population of primary oocytes, as conventional wisdom holds.

### The Evolution of Lifespan

High-predation guppy populations have longer total lifespans because they have significantly longer reproductive lifespans. The differences in longevity of these populations can be visualized with a timeline that is the sum of the mean age at first reproduction, mean duration of the reproductive lifespan, and median post-reproductive lifespan (Figure 4). This visual summary of the results shows graphically that the reason that guppies from high-predation localities live longer is solely because they have longer reproductive lifespans. It thus appears that evolution has shaped that component of the life history that makes a direct contribution to fitness and that, as predicted, the post-reproductive lifespan, which has no impact on fitness, is highly variable and that there are no significant differences among treatment groups in this variable.

**Figure 4:**
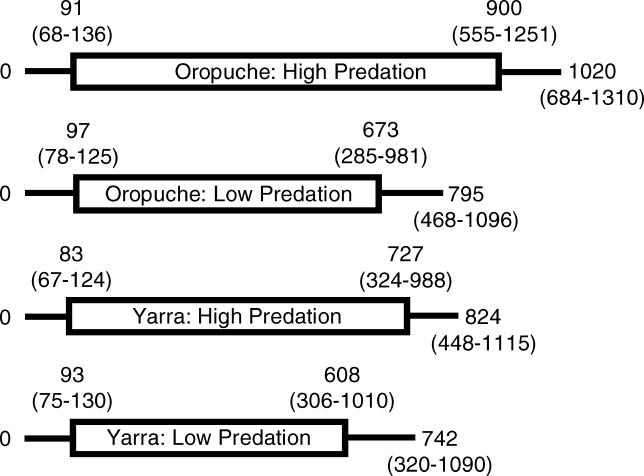
Summary of the Total Lifespan of Guppies, Reported Separately for Each Drainage × Predation Combination The timeline reports the mean age at first reproduction, mean age at last reproduction (mean age at first reproduction plus mean reproductive lifespan), and total lifespan (mean age at first reproduction plus mean reproductive lifespan plus median post-reproductive lifespan). The range of values is reported in parentheses next to each mean and median value. This summary illustrates the overall differences in total lifespan and the fact that these differences are attributable to the duration of the reproductive lifespan alone. Note that we averaged the results for high- and low-food availability to simplify the presentation.

The marginally significant results for drainage of origin and food availability in the logistic regression analysis of post-reproductive lifespan indicate that a larger study could reveal that guppies from the Oropuche drainage and guppies reared on high food availability are more likely to have significant differences in post-reproductive lifespans. In both cases, the higher probability of an extended post-reproductive lifespan is positively correlated with the duration of the reproductive lifespan, which suggests that it increased as a correlate of the increase in reproductive lifespan. This correlation is relevant to our second hypothesis explaining heterogeneity among treatment groups in post-reproductive lifespan, which is that the post-reproductive period might be positively correlated with other components of the life history, regardless of whether or not it contributes to individual fitness. Hendry et al.'s results [21] for anadromous salmon provide a telling contrast to our results. Salmon are a particularly dramatic example of the evolution of resource allocation to reproduction, somatic maintenance, and post-reproductive lifespan because they stop feeding as they migrate from the ocean into their natal streams. Their life history from that point on is like the flight of a ballistic missile, since all activities are fueled by the reserves that they obtained prior to the cessation of feeding. In the population that Hendry et al. studied, there is variation in when females arrive at the breeding ground. Early-arriving females have an advantage in attaining the best breeding sites, but they must also live long enough to protect their nests from late-arriving females. If the nest is not guarded, then the site can be reused, which drastically reduces the survival of her eggs. There is thus positive selection for post-reproductive survival in early-arriving females. These females do indeed live longer after laying their eggs than late-arriving females. They appear to be able to do so because they have an increased retention of fat reserves for somatic maintenance during the guarding period, but do so at the expense of reduced fecundity. It is this tradeoff between reproduction, fat storage, and post-reproductive lifespan that argues for the differences in allocation between early- and late-arriving females being an adaptive feature of the life history. Analyses of neutral genetic variation at microsatellite loci confirm that there is sufficient genetic isolation between the early- and late-arriving cohorts for there to be limited gene flow between them and some adaptive divergence between them.

More generally, it is fair to say that we know far too little about the extent to which natural selection can shape post-reproductive lifespan, largely because too little effort has been invested in comparative, evolutionary studies. Most of the arguments that we are familiar with pertain to humans which, by themselves, represent an unreplicated observation. The comparison between humans and closely related primates [1] is telling because it argues that an extended post-reproductive lifespan is a recent innovation and is not typical of other great apes or other hominids [46]. When combined with Lahdenpera et al.'s demonstration [3] that the post-reproductive lifespan of menopausal woman is strongly associated with their children's reproductive success, then there is a stronger argument that the post-reproductive lifespan of humans is in fact an adaptation. The aggregate of results thus far argues that it is possible to develop predictions about post-reproductive survival in such a comparative framework and thus perform adaptive analyses, as done for so many other aspects of the life history.

## Materials and Methods

We compared the life histories of guppies from high- and low-predation localities in the Yarra and Oropuche drainages of the Northern Range Mountains of Trinidad. Genetic data suggest that the adaptation of guppies to the presence or absence of predators occurred independently in each drainage [29,30]. One high- and one low-predation locality was sampled from each drainage, hence the four localities can be thought of as two paired comparisons between high- and low-predation environments. The subjects of this experiment consisted of the second generation of laboratory-born offspring derived from wild-caught females. The breeding design used to produce these fish used an equal number of grandchildren descended from each wild-caught female. The design thus retained the genetic diversity of the original sample while avoiding inadvertent selection for adaptation to laboratory conditions (details in [33,34]).

Each locality was represented by 30 sibships, and each sibship by two sisters, one reared at high and one at low levels of resource availability; the experiment thus included a total of 240 individuals in eight treatment groups, but after accidental deaths, only 226 were included in the analyses. The fish were measured every other week, and food availability was increased to accommodate growth. All individuals in a given food treatment were given the same amount of food for each 2-wk time interval. Food availability became constant after 9 mo. The high-food treatment received an average of 2.5 times as much food as the low-food treatment.

Food was included as a factor in the design because low-predation localities tend to have lower levels of food availability and hence lower growth rates and smaller asymptotic body sizes in comparison to guppies from high-predation localities. These differences largely disappear when the fish are reared in the lab on similar levels of food availability. The food-availability trajectories were chosen to approximate the average growth rates and asymptotic body sizes of fish from high- versus low-food environments in nature [47]. Partridge and Barton [48] argued that the results of comparative studies of aging in a common environment may be biased if they differ in how well each is adapted to the chosen environment in the lab. Our alternative food levels match the alternative environments experienced in the field and enable us to assess the possibility of this type of adaptation, which would be seen as an interaction between food availability and predation regime. No such interactions were seen in the variables considered in this paper, but since food represented a significant main effect on many dependent variables, it is retained in the presentation of these results. Table 3 includes mean values for asymptotic body size and growth rate that illustrate the consequences of the different food levels for growth. Growth rates are estimated from a fitting of the Von Bertalanffy growth equation to each individual's growth history using Proc NLIN [49].

Each female was isolated in a 7.8-l aquarium when 25 d old. She received a measured volume of food, liver paste in the morning and newly hatched brine shrimp nauplii in the afternoon. Food availability was quantified volumetrically, to the nearest microliter, with a Hamilton micropipette. Females were mated once a week until they gave birth to their first litter of young, then were mated again after the birth of each litter, when they are particularly receptive to mating [50]. All females were maintained for the entirety of their lifespans. The dependent variables include: growth, asymptotic body size, age at maturity, age when each litter was produced, number and size of young in each litter, age at last reproduction, and age at death.

The focus of the current analyses is the division of the lifespan of each individual into the non-overlapping pre-reproductive, reproductive, and post-reproductive segments.

### Post-reproductive lifespan

For each individual, we asked whether the time interval between when it last gave birth and when it died was significantly longer than the interval of time required for that individual to have given birth to its next litter of young. Because individuals vary in the regularity of the intervals between successive litters, we calculated the 90th percentile for the duration of time between successive litters for each individual based on its own reproductive schedule. We chose the 90th percentile as a threshold for classifying individuals as either dying within the time interval when they could have produced an additional litter or as having lived longer than the expected amount of time required to produce another litter of young. If the time interval between the birth of the last litter and death exceeded the 90th percentile interval, then it was judged to have lived beyond the time when it should have given birth to another litter of young and hence was likely to have had a significant post-reproductive lifespan. Dichotomizing the data into those that did or did not live beyond this confidence interval provides a criterion for comparing treatment groups for the probability of significant post-reproductive lifespan. The results were qualitatively the same if we used the 95th percentile as the threshold.

We first compared treatment groups with parametric analyses (SAS, Proc GLM [49]) by dividing each individual's lifespan in to three non-overlapping segments: the age at maturity, the reproductive lifespan (age at last reproduction minus age at maturity), and the post-reproductive lifespan (age at death minus age at last reproduction). The distribution of the first two segments of the life history approximated a normal distribution sufficiently well to justify the use of parametric statistics. The distribution of post-reproductive lifespan did not conform to a normal distribution when either untransformed or log-transformed. Although ANOVAs are robust to non-normality, we also performed a permutation test [51] on the post-reproductive lifespan results to confirm that there were not significant main effects or interactions.

We performed logistic regression analyses on the relationship between post-reproductive lifespan and the different treatment groups (drainage, predation, and food availability). We classified individuals according to whether they died within or outside of the 90th percentile for time between litters. We used the SAS Logistic Regression Procedure [49] to evaluate whether or not the inclusion of drainage, food availability, or predation accounted for significant differences in the probability of having a post-reproductive lifespan.

We extracted similar data on post-reproductive lifespan from Comfort's [7] results for domestic guppies. He reported the age at last reproduction and age at death for 36 fish kept singly or in groups of five. Since he did not report other details of their life history, we were not able to calculate 90th percentile for time between litters, as we did for wild guppies. We could, however, characterize the distribution of post-reproductive lifespans and compare them with data from our laboratory.

## Abbreviations

ANOVA: analysis of variance
